# Biceps tenotomy or tenodesis in association with rotator cuff repair: is there an influence on functional results? A retrospective cohort study

**DOI:** 10.1590/1516-3180.2021.0219.R1.28062021

**Published:** 2022-03-14

**Authors:** Eduardo Angeli Malavolta, Alana Caselato de Sousa, Mauro Emilio Conforto Gracitelli, Jorge Henrique Assunção, Fernando Brandão de Andrade e Silva, Arnaldo Amado Ferreira

**Affiliations:** I MD, PhD. Attending Orthopedic Surgeon, Department of Orthopedics and Traumatology, Hospital das Clinicas HCFMUSP, Faculdade de Medicina, Universidade de Sao Paulo, Sao Paulo, SP, BR.; II MD. Attending Orthopedic Surgeon, Department of Orthopedics and Traumatology, Hospital das Clinicas HCFMUSP, Faculdade de Medicina, Universidade de Sao Paulo, Sao Paulo, SP, BR.; III MD, PhD. Attending Orthopedic Surgeon, Department of Orthopedics and Traumatology, Hospital das Clinicas HCFMUSP, Faculdade de Medicina, Universidade de Sao Paulo, Sao Paulo, SP, BR.; IV MD. Attending Orthopedic Surgeon, Department of Orthopedics and Traumatology, Hospital das Clinicas HCFMUSP, Faculdade de Medicina, Universidade de Sao Paulo, Sao Paulo, SP, BR.; V MD, PhD. Attending Orthopedic Surgeon, Department of Orthopedics and Traumatology, Hospital das Clinicas HCFMUSP, Faculdade de Medicina, Universidade de Sao Paulo, Sao Paulo, SP, BR.; VI MD, PhD. Head of the Shoulder and Elbow Group, Department of Orthopedics and Traumatology, Hospital das Clinicas HCFMUSP, Faculdade de Medicina, Universidade de Sao Paulo, Sao Paulo, SP, BR.

**Keywords:** Tenotomy, Tenodesis, Rotator cuff, Biceps tenotomy, Biceps tenodesis, Rotator cuff repairs, Arthroscopy

## Abstract

**BACKGROUND:**

Instability or tears of the long head of the biceps tendon (LHBT) may be present in more than 35% of rotator cuff repairs (RCR).

**OBJECTIVE:**

To compare clinical results from patients undergoing arthroscopic RCR, according to the procedure performed at the LHBT.

**DESIGN AND SETTING:**

Retrospective cohort study designed at the shoulder and elbow clinic of Instituto de Ortopedia e Traumatologia, Hospital das Clinicas, Faculdade de Medicina, Universidade de Sao Paulo, Brazil.

**METHODS:**

Functional results among patients were compared using the American Shoulder and Elbow Surgeons (ASES) and University of California Los Angeles (UCLA) scales, according to the LHBT approach adopted: no procedure, tenotomy or tenodesis.

**RESULTS:**

We evaluated 306 shoulders (289 patients): 133 underwent no procedure at the LHBT, 77 tenotomy and 96 tenodesis. The ASES scale at 24 months showed no difference (P = 0.566) between the groups without LHBT procedure (median 90.0; interquartile range, IQR 29), tenotomy (median 90.0; IQR 32.1) or tenodesis (median 94.4; IQR 22.7); nor did the UCLA scale (median 33; IQR 7 versus median 31; IQR 8 versus median 33; IQR 5, respectively, P = 0.054). The groups differed in the preoperative functional assessment according to the ASES and UCLA scale, such that the tenodesis group started from higher values. However, there was no difference in pre and postoperative scores between the groups.

**CONCLUSION:**

Tenodesis or tenotomy of the LHBT, in the sample analyzed, did not influence the clinical results from RCR, as assessed using the ASES and UCLA scales.

## INTRODUCTION

Instability or tears of the long head of the biceps tendon (LHBT) may be present in more than 35% of the arthroscopies performed to repair the rotator cuff.^1,2^ Although LHBT disorders may occur in isolation, in most cases involvement of the LHBT is associated with rotator cuff syndrome.^3^

Tenotomy and tenodesis of this tendon are frequently performed during shoulder arthroscopy.^3^ The low number of studies comparing these techniques has been highlighted in published meta-analyses,^4–6^ and in one of them 50% of the studies only present level IV evidence.^4^ The results pointed towards better functional results through tenodesis, but no minimally significant clinical difference was reached.^5,6^ However, no analysis on confounding factors was performed in these meta-analyses,^6^ and the samples may have been subject to selection bias, especially in non-randomized studies. In addition, most of the published studies compared the results obtained through tenodesis and tenotomy, and only a few reports included a control group of patients without biceps procedures.^7^

## OBJECTIVE

The aim of this study was to compare the clinical results from patients undergoing rotator cuff repair, divided into three groups according to the procedure performed at the LHBT: control group (without any LHBT approach), tenotomy or tenodesis.

## METHODS

### Design, place and dates

We performed a retrospective cohort study, with data collected prospectively, comparing the functional results between three groups of patients according to the intraoperative approach to the LHBT that had been used: no procedure, tenotomy or tenodesis. The procedures were performed between 2014 and 2017, in a single institution, by three different surgeons.

The local institutional review board approved the study: the Ethics Committee for Review of Research Projects (“CAPPesq”) of the Clinical Board of the Hospital das Clinicas HCFMUSP, Faculdade de Medicina, Universidade de Sao Paulo, under research protocol number 2.133.213, dated June 22, 2017.

### Eligibility criteria

We included patients who underwent arthroscopic rotator cuff repair and had preoperative magnetic resonance imaging, without the use of intra-articular contrast, in a device rated at 1.5T or higher, regardless of tendon thickness, retraction or fatty degeneration. Patients with irreparable or partially repaired rotator cuff tear, tear of the subscapularis alone, complete tear of the LHBT, rotator cuff arthropathy, moderate or severe glenohumeral arthrosis (as described by Samilson and Prieto)^8^ or previous shoulder surgery were excluded. Patients who had not undergone pre or postoperative functional evaluation were also excluded.

### Outcome

The primary outcome was the evaluation on the American Shoulder and Elbow Surgeons (ASES)^9,10^ and University of California Los Angeles (UCLA)^11,12^ scales at a follow-up conducted 24 months after the operation. As a secondary outcome, we assessed the influence of sex, age, dominance, smoking, diabetes, previous injection, traumatic tear, supraspinatus tear pattern, subscapularis repair, fatty degeneration of rotator cuff muscles, acromioplasty and lateral clavicle resection on the clinical results from the LHBT procedure.

### Variables analyzed

The following variables were evaluated:

- Variables relating to patients: age, sex, involvement of the dominant side, smoking, diabetes, previous injection and previous trauma to the affected shoulder.
- Variables relating to the lesion: thickness of the supraspinatus tear (partial or full thickness), retraction of the supraspinatus tear (< 30 mm or ≥ 30 mm) and degree of fatty infiltration of the rotator cuff muscles, as described by Fuchs et al.^13^
- Variables relating to the procedure: subscapularis repair, acromioplasty and distal resection of the clavicle.
- Variables relating to the intraoperative approach towards the LHBT: no procedure, tenotomy or tenodesis. In cases of tenodesis, we specified the method of fixation and site of tenodesis.

All variables referring to the lesion, with the exception of fatty degeneration, were determined by means of arthroscopic inspection. The clinical evaluations using the ASES^9,10^ and UCLA^11,12^ scales were performed one week before and 24 months after the surgical procedure.

### Arthroscopy

The procedures were performed under general anesthesia in association with interscalene brachial plexus block. We positioned the patients on a beach chair or in lateral decubitus, depending on the surgeon’s preference, and conventional portals were used. The inspection was performed using a 30° arthroscope positioned in the posterior portal. Using a probe, the LHBT was palpated and mobilized, looking for signs of instability, through the “ramp test”,^14^ as well as any injuries to its substance or insertion. The variables described above were inspected in a standardized manner.

The indications for LHBT procedures were subluxation or dislocation, partial lesions affecting more than 25% of the thickness or superior labrum lesions of types 2, 3 or 4. For patients aged 60 years or older, tenotomy was performed. Tenodesis was performed on younger or active patients or on those with a body mass index (BMI) below 25, regardless of age. Tenotomy was performed close to the origin of the glenoid labrum. Tenodesis, when indicated, was performed using anchors. In the procedure, one of the anchor sutures used to repair the subscapular or supraspinatus could be used, or an additional anchor positioned in the bicipital groove could be used.

The rotator cuff repair was performed using a simple row technique. Acromioplasty was performed according to the surgeon’s preference, and distal resection of the clavicle was performed in patients with symptomatic arthritis. After surgery, patients remained immobilized through use of a sling for six weeks.

Passive shoulder movements were started in the third week, and active shoulder movements were started after the sling was removed. Strengthening started at 12 weeks. Movements of the hand, wrist and elbow were allowed from day one.

### Statistical analysis

We subjected continuous variables to assessment of normality, using the Shapiro-Wilk test, and assessment of homogeneity, using the Levene test.

Categorical variables were described in terms of absolute and percentage values. Continuous variables were expressed as the median and interquartile range (IQR). The general characteristics of the sample were compared between groups using the chi-square test (categorical variables) or the Kruskal-Wallis test (continuous variables).

Comparisons between pre and postoperative times for each group, according to the measurements on the ASES and UCLA scales, were performed using the Wilcoxon test. The comparison between functional results before surgery and at 24 months, according to the approach taken to the LHBT, was performed using the Kruskal-Wallis test, with Bonferroni post-hoc correction.

Multiple regression analysis was performed with the objective of identifying confounding factors in the final result, and this analysis including all variables that presented P < 0.2.

We used the SPSS software (version 21.0; IBM, Armonk, New York, United States) for data analysis. A 5% significance level was used.

## RESULTS

During the study period, we performed 399 arthroscopies to treat rotator cuff tears. Patients who underwent debridement (11), subscapularis repair alone (5), reoperations (3) or rotator cuff partial repair (34), patients with complete tears of the LHBT (19) and those without clinical information (21) were not included in the analysis. Thus, the sample analyzed consisted of 306 shoulders (289 patients), among which 133 shoulders did not undergo any procedure on the long head of the biceps, while 77 underwent tenotomy and 96 underwent bicipital tenodesis. Tenodesis was performed on at the supraspinatus repair anchor in the cases of 62 shoulders and at the subscapular anchor in 24 cases. Tenodesis was performed in the bicipital groove in 10 shoulders.

The variables relating to the patients demonstrated that the groups differed in terms of age and sex (P = 0.022 and P < 0.001, respectively). Cases involving tenotomy presented higher mean age and were predominantly among women, while cases involving tenodesis were predominantly among men. There were no differences in the other analyses (Table 1).

**Table 1.** Variables relating to the patients

	Biceps procedure						
	None (n = 133)		Tenotomy (n = 77)		Tenodesis (n = 96)		P
**Age, years [median, IQR]**	53	10	62	12	56	9	0.022
**Sex [n (%)]**							
Male	53	39.8	17	22.1	58	60.4	< 0.001
Female	80	60.2	60	77.9	38	39.6	
**Dominant side affected [n, %]**							
Yes	95	71.4	56	72.7	69	71.9	0.980
No	38	28.6	21	27.3	27	28.1	
**Smoking [n, %]**							
Smoker	16	12.0	9	11.7	15	15.6	0.768
Former smoker	26	19.5	11	14.3	17	17.7	
Never smoked	91	68.4	57	74.0	64	66.7	
**Diabetes [n, %]**							
Yes	18	13.5	13	16.9	11	11.5	0.586
No	115	86.5	64	83.1	85	88.5	
**Previous injection [n, %]**							
Yes	24	18.0	11	14.3	13	13.5	0.604
No	109	82.0	66	85.7	83	86.5	
**Traumatic tear [n, %]**							
Yes	11	8.3	8	10.4	11	11.5	0.712
No	122	91.7	69	89.6	85	88.5	

IQR = interquartile range.

The variables relating to the injury and the procedure showed statistically significant differences in all analyses, except for distal clavicle resection. The group without any procedure at the LHBT had fewer full-thickness tears of the supraspinatus, less retraction of the supraspinatus tear, lesser degree of fatty degeneration and a lower index of subscapularis repair. The tenotomy group had more retracted lesions and more fatty degeneration, compared with the tenodesis group. These data can be seen in Table 2.

**Table 2.** Variables relating to the lesion and the procedure

	Biceps procedure						
	None (n = 133)		Tenotomy (n = 77)		Tenodesis (n = 96)		P
**Supraspinatus tear [n, %]**							
Partial thickness	28	21.1	4	5.2	4	4.2	< 0.001
Full thickness	105	78.9	73	94.8	92	95.8	
**Retraction of the supraspinatus [n, %]**							
< 30 mm	119	89.5	46	59.7	74	77.1	< 0.001
≥ 30 mm	14	10.5	31	40.3	22	22.9	
**Subscapularis repair [n, %]**							
Yes	19	14.3	44	57.1	54	56.3	< 0.001
No	114	85.7	33	42.9	42	43.8	
**Fuchs classification (supraspinatus) [n, %]**							
Grade I	123	92.5	51	66.2	84	87.5	< 0.001
Grade II	9	6.8	20	26.0	12	12.5	
Grade III	1	0.8	6	7.8	0	0.0	
**Fuchs classification (infraspinatus) [n, %]**							
Grade I	122	91.7	57	74.0	86	89.6	0.002
Grade II	11	8.3	16	20.8	9	9.4	
Grade III	0	0.0	4	5.2	1	1.0	
**Fuchs classification (subscapularis) [n, %]**							
Grade I	130	97.7	65	84.4	93	96.9	< 0.001
Grade II	3	2.3	11	14.3	3	3.1	
Grade III	0	0.0	1	1.3	0	0.0	
Acromioplasty [n, %]							
Yes	114	85.7	50	64.9	81	84.4	0.001
No	19	14.3	27	35.1	15	15.6	
**Distal clavicular resection [n, %]**							
Yes	6	4.5	1	1.3	0	0.0	0.063
No	127	95.5	76	98.7	96	100.0	

The multiple regression analysis showed that only age proved to be an independent factor for the postoperative clinical outcome (P = 0.007), such that older patients correlated with better functional results. These data are shown in Table 3.

**Table 3.** Multiple regression analysis to control for confounding factors

	Coefficient	95% confidence interval		P
		Lower	Upper	
Sex	−0.082	−8.692	1.726	0.189
Age	0.170	0.116	0.711	0.007
Supraspinatus tear	0.023	−6.032	9.064	0.693
Supraspinatus retraction	−0.077	−10.816	2.985	0.265
Subscapularis repair	−0.089	−8.998	1.245	0.137
Fuchs classification (supraspinatus)	−0.024	−8.416	6.111	0.755
Fuchs classification (infraspinatus)	−0.088	−12.026	2.792	0.221
Fuchs classification (subscapularis)	0.072	−3.984	15.989	0.238
Acromioplasty	0.030	−4.842	8.041	0.625
Distal clavicular resection	−0.015	−17.934	13.720	0.793
Preoperative ASES score	0.106	−0.014	0.250	0.079

ASES = American Shoulder and Elbow Surgeons.

All three groups improved significantly through the procedure, according to the two scales used (P < 0.001). The ASES scale at 24 months showed no significant difference (P = 0.566) between the groups without LHBT procedure (median 90.0; IQR 29), tenotomy (median 90.0; IQR 32.1) and tenodesis (median 94.4; IQR 22.7). Likewise, the UCLA scale did not differ between the groups (median 33; IQR 7 versus median 31; IQR 8 versus median 33; IQR 5, respectively, P = 0.054). The groups differed significantly in the preoperative functional assessment, according to both the ASES scale (P = 0.017) and the UCLA scale (P = 0.008), and the tenodesis group started from higher values. However, there was no difference in pre and postoperative scores between groups (P = 0.642 and 0.371, respectively). These data are shown in Table 4.

**Table 4.** ASES and UCLA scores according to biceps procedure

	Biceps procedure						P
	None (n = 133)		Tenotomy (n = 77)		Tenodesis (n = 96)		
	Median	IQR	Median	IQR	Median	IQR	
**ASES**							
Preoperative	38.3	25.5	34.6	26.7	44.8	25.7	0.017*
24 months	90	29	90	32.1	94.4	22.7	0.566
Difference***	43.9	41.6	45	44	43.4	29.9	0.642
**UCLA**							
Preoperative	14	6	14	6	15	8	0.008**
24 months	33	7	31	8	33	5	0.054
Difference***	17	9	16	8	15	8	0.371

ASES = American Shoulder and Elbow Surgeons; UCLA = University of California Los Angeles; *Post-hoc test: tenotomy vs. tenodesis, P = 0.017; other comparisons, P > 0.05; **Post-hoc test: tenotomy versus tenodesis, P = 0.033; none versus tenodesis, P = 0.014; other comparisons, P > 0.05; ***Difference between postoperative and preoperative scores.

We performed a subgroup analysis, categorizing patients by age, according to the result from the multivariate analysis. From the ASES scale, patients aged 60 years or older had similar scores in the preoperative evaluation (medians for the control group 36.1; tenotomy group 27.1; tenodesis group 50; P = 0.105) and at 24 months (medians of 99, 93.9 and 94.7, respectively; P = 0.247). Likewise, among the patients under the age of 60 years, the scores were similar between the groups in the preoperative assessment (medians of 38.9, 32.4 and 42.8, respectively; P = 0.096) and in the assessment at 24 months (medians of 88.2, 84.4 and 93.2, respectively; P = 0.224). In the evaluation using the UCLA scale, patients under 60 years of age who underwent tenodesis showed slightly higher postoperative results (median of 33), compared with patients in the control group (32) and in the tenotomy group (31), with a statistically significant difference (P = 0.027). In the preoperative assessment, the difference also favored the tenodesis group (median of 15), compared with the groups without the procedure (13.5) and tenotomy (12), with a statistically significant difference (P = 0.024). In the subgroup of patients older than 60 years, there were no statistically significant differences in the preoperative evaluation using the UCLA scale (medians of 14, 14 and 17, respectively; P = 0.193), or in the 24-month evaluation (35, 32 and 35; P = 0.248).

The location of LHBT tenodesis did not influence the results. The 86 patients who underwent intra-articular tenodesis had a median score of 94.4 (IQR 24.1) using the ASES scale and 33.5 (IQR 6) using the UCLA scale, while the 10 patients who underwent tenodesis in the bicipital groove had a median score of 93.2 (IQR 20.4) and 33 (IQR 4), respectively. The P values were 0.590 for the ASES scale and 0.695 for the UCLA scale.

## DISCUSSION

Our results showed that, at 24 months, the functional results from arthroscopic rotator cuff repair did not differ between patients undergoing tenotomy, tenodesis or no procedure at the LHBT. These results are in line with those presented by Kukkonen et al.,^7^ in a cohort study that, similarly to ours, compared three groups of patients according to the procedure performed at the LHBT, including a control group. However, in our series, the patients started from statistically different scores at the baseline, such that the tenotomy group had a lower score than the tenodesis group before surgery. Nonetheless, this difference did not reach clinical relevance, and its detection may have been due to our larger sample (about three times more patients underwent procedures at the LHBT). Those authors had a less detailed baseline than ours, and they evaluated their sample only according to sex and age, without analyses on other possible confounding factors. Several other factors such as comorbidities and associated lesions and procedures can influence the results, especially due to the fact that procedures relating to the LHBT are performed together with rotator cuff repair in most cases.

It is important to highlight that although the patients presented statistically significant differences regarding their preoperative scores (such that the tenodesis group started with higher baseline scores), the values found at the end of 24 months did not differ between the groups; nor was there a difference between the pre and postoperative states. The lack of significant difference between the groups regarding the improvement obtained (calculated as the difference between pre and postoperative states) was maintained even after analyzing the subgroups according to age.

In a recent systematic review comparing the effects of tenodesis and tenotomy, Na et al.^6^ observed that there was a statistically significant difference favorable to the tenodesis group, according to the Constant-Murley score (96.5 versus 95.6). However, some points should be highlighted. The difference found did not reach clinical relevance^15^ and, in addition, the study did not evaluate the baseline score, which may be higher in the tenodesis group, as shown in our study, which may be a confounding factor for the analysis. Most of the published comparative studies have not found any statistically significant difference regarding clinical scales,^7,16,17,18,19,20,21,22,23,24,25^ and those that did demonstrate a statistical difference did not reach clinical relevance.^20,26^

Our study did not evaluate complications relating to procedures at the LHBT. Popeye’s sign occurs in 3%-6% of the cases undergoing tenodesis, and in 9%-20% of those undergoing tenotomy, according to randomized studies.^22–24^ In a systematic review of level II clinical evidence, Na et al.^6^ demonstrated a statistically significant difference, such that 24% of the patients in the tenotomy group showed Popeye’s sign, compared with 9% in the tenodesis group. Other complications, such as postoperative pain level, presence of cramping and elbow flexion strength and forearm supination, have not been found to differ between tenotomy and tenodesis groups.^6^ Furthermore, arm deformity is not a source of concern for most patients.^16,17^ Among 24 patients with Popeye’s sign, Boileau et al.^17^ noted that only 16 noticed the deformity and none of the patients cared about it. Biz et al.,^16^ similarly, reported that only 25% of the patients with Popeye’s sign had noticed the deformity.

In our series, procedures at the LHBT were necessary in 56% of the arthroscopies, i.e. a higher proportion than the 40% reported by other authors.^7,27^ This was probably due to inclusion of a greater number of patients with tears not restricted to the supraspinatus.

Our results, which showed similarity between those from tenodesis performed with an anchor next to the rotator cuff and those performed in the bicipital groove with an anchor, should be viewed with caution, given the small sample of patients in the second group. Franceschetti et al,^28^ in a randomized study, observed that the clinical results from subpectoral tenodesis were superior to those from high tenodesis with anchors. However, those authors performed open subpectoral tenodesis using a screw, whereas they performed high tenodesis using anchors and arthroscopically. Thus, it is not possible to say whether the difference found was due to the method or to the site of fixation. Most studies have aimed to compare tenodesis with tenotomy, and not to make comparisons between different types of tenodesis.^5,6,23^

Our study had some limitations. First, it was a retrospective cohort study, with the biases inherent to this design. It is important to highlight that the groups were heterogeneous regarding baseline characteristics and preoperative functional assessments. To reduce bias, we performed multivariate and subgroup analysis. Future randomized studies may bring more knowledge on this topic. It is noteworthy that, despite the initial differences, the groups converged to similar functional results at the end of the follow-up.

In addition, we did not perform postoperative resonance imaging (MRI), and structural analysis on the cuff repair or tenodesis was not possible. However, it is known that the functional results do not reach any clinically important difference between patients with and without structural integrity of the rotator cuff,^29^ and most studies that have evaluated the results of procedures at the LHBT did not carry out this analysis.^6^ Furthermore, although we applied scales that are widely used for functional assessment of the shoulder (ASES and UCLA), we did not evaluate specific physical findings of involvement of the LHBT in the physical examination, such as anterior shoulder pain or the incidence of Popeye’s sign.

Our sample was more heterogeneous than that of other authors,^7^ including massive rotator cuff tears and involvement not restricted to the supraspinatus. It is already known that the dimension of the rotator cuff tear correlates with LHBT lesions, and this factor may have biased our results.^30^ On the other hand, this approach increased the external validity of the data.

It is also worth noting that our data are applicable only to suprapectoral tenodesis with anchors, and cannot be generalized to other techniques. In addition, the surgical technique used was not standardized, and included different tenodesis sites and use of an anchor for the LHBT independently or in association with rotator cuff repair. Most comparative studies have evaluated tenodesis with anchors,^7,19–22,25^ and few have evaluated use of interference screws.^17,24^

Among the favorable points regarding our study, we can mention the inclusion of the control group in which no surgical approach was performed at the LHBT. This strategy was previously only used by Kukkonen et al.^7^ and Godenèche et al.,^26^ to the detriment of the other cohorts^16–21,26^ or randomized studies.^22–24^ Our sample was quite robust, and superior to that of most other comparative studies,^16–24,26^ even though we only considered patients who underwent one of the biceps procedures. Furthermore, all the patients underwent preoperative MRI. We made a detailed description of the baseline, associated with multivariate regression, in order to search for factors that might confound the clinical outcome. This statistical approach, which is important in cohort studies, has only been implemented in a few studies.^17,26^ Lastly, our postoperative clinical evaluation was carried out at a standardized time, at 24 months after the operation, by a research assistant who did not participate otherwise in the study, which therefore reduced the measurement bias.

Thus, according to our results and supported by the current literature,^5,6,23^ we concluded that the procedure performed at the LHBT did not influence the final result when performed in association with rotator cuff repair. Future research analyzing the influence of the LHBT on functional results and on patient satisfaction and quality of life, along with development of clinical scales for assessing this tendon and studies comparing different types of tenodesis, are needed for better understanding of the role of different approaches to the biceps with regard to the clinical results.

## CONCLUSION

The choice between tenodesis and tenotomy of the long head of the biceps, in the sample analyzed, did not influence the clinical results from rotator cuff repair evaluated using the ASES and UCLA scales.
